# Exploring the pivotal variables of tongue diagnosis between patients with chronic kidney disease and health participants

**DOI:** 10.3389/fdata.2024.1443646

**Published:** 2025-01-03

**Authors:** Po-Chi Hsu, Jia-Ming Chen, Chia-Chu Chang, Yu-Jun Chang, Ping-Fang Chiu, John Y. Chiang, Lun-Chien Lo

**Affiliations:** ^1^School of Chinese Medicine, China Medical University, Taichung, Taiwan; ^2^Department of Chinese Medicine, China Medical University Hospital, Taichung, Taiwan; ^3^Department of Traditional Chinese Medicine, Changhua Christian Hospital, Changhua, Taiwan; ^4^Graduate Institute of Chinese Medicine, China Medical University, Taichung, Taiwan; ^5^Division of Nephrology, Department of Internal Medicine, Kuang Tien General Hospital, Taichung, Taiwan; ^6^Big Data Center, Epidemiology and Biostatistics Center, Changhua Christian Hospital, Changhua, Taiwan; ^7^Nephrology Division, Department of Internal Medicine, Changhua Christian Hospital, Changhua, Taiwan; ^8^Department of Computer Science and Engineering, National Sun Yat-Sen University, Kaohsiung, Taiwan; ^9^Department of Healthcare Administration and Medical Informatics, Kaohsiung Medical University, Kaohsiung, Taiwan

**Keywords:** Traditional Chinese Medicine (TCM), tongue diagnosis, automatic tongue diagnosis system (ATDS), chronic kidney disease (CKD), renal function

## Abstract

**Introduction:**

Chronic kidney disease (CKD) is a significant global health problem associated with high morbidity and mortality rates. Traditional Chinese Medicine (TCM) utilizes tongue diagnosis to differentiate symptoms and predict prognosis. This study examines the relationship between tongue characteristics and CKD severity using an automatic tongue diagnosis system (ATDS), which captures tongue images non-invasively to provide objective diagnostic information.

**Methods:**

This cross-sectional, case-control study was conducted from July 1, 2019, to December 31, 2021. Participants were divided into three groups based on estimated glomerular filtration rate (eGFR): control (eGFR > 60 ml/min/1.732), CKD stage 3 (30 ≤ eGFR < 60 ml/min/1.732), and CKD stage 4–5 (eGFR < 30 ml/min/1.732). Tongue images were analyzed using ATDS to extract nine primary features: tongue shape, color, fur, saliva, fissures, ecchymosis, tooth marks, and red dots. Statistical analyses included non-parametric methods and ordinal logistic regression.

**Results:**

This study revealed that significant differences in the fur thickness, tongue color, amount of ecchymosis, and saliva among three groups. Ordinal logistic regression indicated that pale tongue color (OR: 2.107, *P* < 0.001), bluish tongue color (OR: 2.743, *P* = 0.001), yellow fur (OR: 3.195, *P* < 0.001), wet saliva (OR: 2.536, *P* < 0.001), and ecchymoses (OR: 1.031, *P* = 0.012) were significantly associated with increased CKD severity. Additionally, each red dot and tooth mark decreased the odds of severe CKD.

**Conclusion:**

Tongue features such as paleness, wet saliva, yellow fur, and ecchymosis are prevalent in CKD patients and can serve as early clinical indicators of the disease. This study demonstrates that TCM tongue diagnosis, facilitated by ATDS, is a valuable, non-invasive method for identifying CKD and distinguishing its stages.

## 1 Introduction

Chronic kidney disease (CKD) is a progressive condition affecting more than 10% of the global population, making it a significant public health concern (Kovesdy, 2022). CKD is characterized by a glomerular filtration rate (GFR) of <60 mL/min/1.73 m^2^ or the presence of other indicators of kidney damage, such as imaging abnormalities or albuminuria, particularly comorbidity with hypertension and diabetes (Webster et al., 2017; Matsushita et al., 2015). CKD progresses from an asymptomatic early stage to debilitating later stages, characterized by symptoms such as fatigue, nausea, and swelling, primarily due to complications like anemia and chronic inflammation (Evans et al., 2022; Charles and Ferris, 2020). As CKD progresses to end-stage renal disease (ESRD), life-sustaining treatments such as dialysis or kidney transplantation become necessary (KDIGO Group, 2020).

The growing prevalence of CKD raises concerns about the cost and health-related quality of life (HRQoL) burden of patients (Honeycutt et al., 2013; Pergola et al., 2019). There is a significant disparity in the evidence about direct and indirect costs attributed to CKD and ESRD, with the most complete evidence concentrated on direct health care costs of patients with advanced to the later stages of CKD (Nichols et al., 2020; Wang et al., 2016). Therefore, it is essential to find effective and conservative treatment methods to slow progression and reduce complications of CKD.

The diagnosis in TCM is based on four main approaches, including inspection, auscultation and olfaction, inquiry, and pulse diagnosis (Tian et al., 2023). Tongue diagnosis, serving as a vital non-invasive tool to provide useful clinical information, plays a crucial role in TCM. The tongue is considered to reflect the physiological and pathological condition of the body, as well as the degree and progression of the disease, through the meridians that connect the tongue to the internal organs (Jiang et al., 2012). By observing tongue features, TCM practitioners can probe qi-blood, yin-yang disorders which are important in treatment selection and prognosis (Feng et al., 2023). Clinically, doctors observe tongue features, such as tongue color and shape, fur color and thickness, and the amount of saliva, to help deduce the primary pattern of a patient (Lo et al., 2012). However, tongue inspection is often biased by subjective judgment, which originates from personal experience, knowledge, diagnostic skills, thinking patterns, and color perception. The inconsistency of subjective diagnosis can be improved by using the development of validated instruments.

The automatic tongue diagnosis system (ATDS) has shown high consistency and can provide objective and reliable information and analysis of tongue characteristics, facilitating doctors to make effective observations and diagnoses of specific diseases (Lo et al., 2012). In recent years, several researches have applied case-control methodology to explore the association between tongue characteristics and specific diseases, including breast cancer (Hsu et al., 2021), type 2 diabetes (Hsu et al., 2016), ischemic stroke (Huang et al., 2022), metabolic syndrome (Lee et al., 2016), and CKD (Chung et al., 2023), in the hope of deriving discriminating tongue features to distinguish individuals with/without the specific disease through a non-invasive means in the early stage. This research aims to identify distinctive tongue features associated with CKD, offering a non-invasive diagnostic tool for early detection and management.

## 2 Methods

### 2.1 Study design

We conducted a cross-section, case-control, and observational study from July 1, 2019 to December 31, 2021. Participants were divided into the following three groups according to the status of their renal function: control group (eGFR > 60 ml/min/1.73^2^), CKD stage 3 group (30 ml/min/1.73^2^ < eGFR < *60* ml/min/1.73^2^) and CKD stage 4–5 group without dialysis (eGFR < 30 ml/min/1.73^2^). Participants were enrolled in the control group without evidence of kidney damage, such as albuminuria or abnormal findings on renal imaging. Subjects with any of the following conditions will be excluded: cancer; acute infection; unable to protrude the tongue stably; risk of temporomandibular joint dislocation (Chen et al., 2021). This study has been reviewed and approved by the Institutional Review Board (IRB) of Changhua Christian Hospital (CCH), Taiwan (IRB Nos. 190404 and 140704). This trial was registered with the National Institute of Health Clinical Trial Registry (clinicaltrials.gov; NCT04708743).

### 2.2 Participants

In this case-control study, we utilized data from a previous study to identify 340 healthy participants, excluding 22 subjects due to insufficient data, spanning from January 1, 2012, to December 31, 2016 (Hsu et al., 2019). We initially identified 370 patients with CKD stage 3 and 111 patients with CKD stage 4–5. Tongue images for all participants, including the control group, CKD stage 3 group, and CKD stage 4–5 group, were collected using the validated Automatic Tongue Diagnosis System (ATDS), which automatically extracted the corresponding tongue features.

### 2.3 Data collection

Information including demography, body mass index (BMI, kg/m^2^), CKD staging, estimated glomerular filtration rate (eGFR) and blood urea nitrogen (BUN) were collected for each subject. The duration of CKD history, comorbidity and complication in patient with CKD were also collected. Tongue images were collected for each subject to further derive the relevant tongue features of every participant. All personal details and photographs of subjects recruited were encrypted to ensure confidentiality.

### 2.4 Automatic tongue diagnosis system

We analyzed the data from the subjects and the tongue features using the ATDS. The ATDS was specifically developed to capture tongue images and automatically extract features reliably, aiding TCM practitioners in diagnosis. The effectiveness of the ATDS lies in its ability to segment the tongue region and extract tongue features automatically. This ensured that all tongue images were captured using the same hardware and software configuration, providing a consistent foundation for the data collection process. The ATDS performs three major functions: image capturing and color calibration, tongue area segmentation, and tongue feature extraction. These processes are seamlessly integrated within the system.

### 2.5 Outcome measures

Nine primary tongue features were extracted using the ATDS: tongue color, tongue shape, saliva, tongue fur, fur thickness, tongue fissure, ecchymosis, tooth marks, and red dots. The extracted features are detailed as follows:

1. Tongue color: includes slightly white, slightly red, red, dark red, and dark purple.
2. Tongue shape: includes shape (thin and small, moderate, fat and large) and tongue body position (normal, tilted to the left, tilted to the right).
3. Saliva: total area and amount of saliva categorized as none, little, normal, or excessive.
4. Tongue fur: includes fur color (white, yellow, dyed), amount, average covering area, maximum covering area, minimum covering area, and degree of thickness (none, thin, thick).
5. Fur thickness: degree of thickness (thin or thick) and the percentage of thick tongue fur.
6. Tongue fissure: includes the number of fissures, average covering area, shortest length, and longest length.
7. Ecchymosis: includes the number of ecchymoses, average covering area, maximum covering area, and minimum covering area.
8. Tooth marks: includes the number of tooth marks, average covering area, maximum covering area, and minimum covering area.
9. Red dots: includes the number of red dots, average covering area, maximum covering area, and minimum covering area.

### 2.6 Data analysis

The tongue features of the participants were extracted using ATDS, and cross compared with the kidney injury severity groups to see if there was a linear correlation between them. A trend analysis was categorized by their eGFR into three groups: eGFR ≥ 60 ml/min/1.732 (control group), 30 ≤ eGFR < 60 ml/min/1.732 (CKD stage 3), and eGFR < 30 ml/min/1.732 (CKD stage 4–5). Data are expressed as number, percentage, median, or interquartile range (IQR, 25th−75th percentile) where appropriate. Since the distribution of tongue features was non-normal, non-parametric statistics were employed for univariate analysis. The Jonckheere-Terpstra test was used for continuous variables, and the chi-square test for trend was used for categorical variables to examine the association between tongue features and kidney injury severity. To identify tongue features predictive of renal impairment, we performed an ordinal logistic regression analysis, adjusting for potential confounders. All potential predictors associated with renal impairment were assessed, and odds ratios (ORs) with corresponding 95% confidence intervals (CIs) were calculated. All data were analyzed using the IBM SPSS Statistics for Windows, Version 22.0 (IBM Corp., Armonk, NY). Statistical significance was set at *p* < 0.05.

## 3 Results

**Figure 1:**
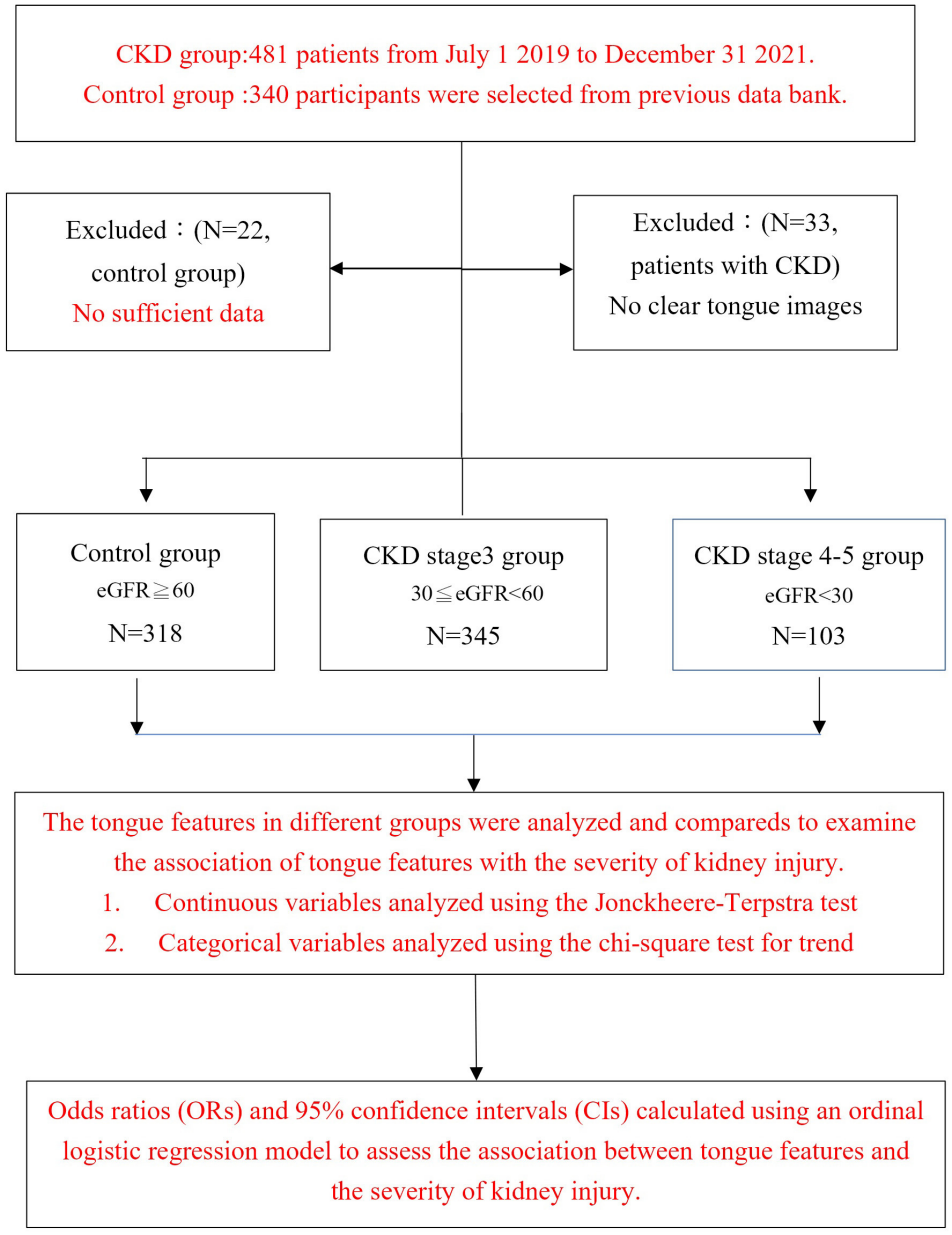
Exploratory flow chart of participant selection for chronic kidney disease and associated tongue diagnosis study.

In this case-control study, we utilized data from a previous study to identify 340 healthy participants, excluding 22 subjects due to insufficient data. From July 1, 2019, to December 31, 2021, 481 patients with chronic kidney disease (CKD) underwent TCM tongue diagnosis. Thirty-three CKD patients were excluded due to unclear tongue images. The final study population consisted of 318 healthy participants (eGFR ≥ 60 ml/min/1.732), 345 patients of stage 3 CKD (30 ≤ eGFR < 60 ml/min/1.732), and 103 patients of stage 4–5 CKD (eGFR < 30 ml/min/1.732). As shown in Figure 1, the exploratory flow chart for CKD-associated tongue diagnosis variates.

This study applied a non-parametric trend test and the demographic and clinical characteristics of the participants were summarized in Table 1. The median age increased with the severity of CKD, with median ages of 63, 65, and 65 years for the control, CKD stage 3, and CKD stage 4–5, respectively (*p* = 0.005). Hemoglobin levels decreased significantly with the severity of CKD (*p* < 0.001). Fasting glucose levels increased significantly with the severity of CKD (*p* < 0.001). Total cholesterol and triglyceride levels exhibited significant variations across the groups, with total cholesterol generally decreasing and triglycerides increasing as renal function worsened (*p* < 0.001 and *p* = 0.030, respectively). Levels of uric acid, blood urea nitrogen (BUN), and creatinine increased significantly with worsening renal function (*p* < 0.001). The proportion of participants with thick tongue fur increased significantly as eGFR level worsened (*p* < 0.001). The numbers of ecchymoses, teeth marks, and red dots decreased significantly with the severity of CKD (*p* < 0.001).

**Table 1:** Demographic and clinical characteristics of study participants with statistical comparison across CKD stages.

	**eGFR** ≧**60**				**30** ≦**eGFR**<**60**				**eGFR**<**30**				***P*-value**	
	***N***	**Median**	**Q**_1_	**Q**_3_	***N***	**Median**	**Q**_1_	**Q**_3_	***N***	**Median**	**Q**_1_	**Q**_3_		
Age	318	63.00	55.00	69.00	345	65.00	56.00	72.00	103	65.00	59.00	74.00	0.005	↑
Systolic pressure	318	126.00	117.00	139.00	329	121.00	112.00	129.00	98	124.00	115.00	136.00	0.002	↓
Diastolic pressure	318	78.00	71.00	84.00	329	73.00	69.00	75.00	98	73.00	69.00	76.00	< 0.001	↓
Body Mass Index	318	23.50	21.40	25.40	120	23.42	21.14	26.02	40	23.36	20.47	26.35	0.684	
Hemoglobin	127	13.80	13.00	14.80	229	12.40	11.10	13.50	82	10.85	9.10	12.20	< 0.001	↓
Glucose (AC)	318	96.00	90.00	103.00	148	105.00	93.00	133.50	41	114.00	94.00	132.00	< 0.001	↑
Total cholesterol	318	195.00	174.00	217.00	152	168.00	143.50	200.00	41	164.00	145.00	184.00	< 0.001	↓
Triglyceride	318	92.00	62.00	136.00	151	107.00	66.00	151.00	44	103.50	63.50	175.00	0.030	↑
Uric acid	114	5.30	4.40	6.00	89	6.40	5.10	7.50	38	6.35	5.40	7.70	< 0.001	↑
BUN	59	12.00	10.00	15.00	127	15.00	12.00	19.00	88	23.00	16.00	40.00	< 0.001	↑
Creatinine	318	0.75	0.63	0.90	345	0.96	0.76	1.20	103	1.59	1.10	2.55	< 0.001	↑
eGFR	318	89.21	77.37	103.59	345	48.54	40.26	55.01	103	22.62	16.86	26.03	< 0.001	↓
Thick tongue fur (%)	313	52.00	38.00	64.00	337	62.00	44.00	81.00	99	64.00	46.00	84.00	< 0.001	↑
Fissures	318	4.00	0.00	11.00	345	5.00	0.00	12.00	103	4.00	0.00	14.00	0.103	
Ecchymoses	318	2.00	0.00	6.00	345	1.00	0.00	4.00	103	0.00	0.00	2.00	< 0.001	↓
Teeth marks	318	4.00	0.00	6.00	345	3.00	0.00	5.00	103	3.00	0.00	5.00	0.003	↓
Red dots	318	59.00	32.00	107.00	345	13.00	3.00	34.00	103	7.00	1.00	25.00	< 0.001	↓

Q_1_, percentile 25; Q_3_, percentile 75; *P*-value by Jonckheere-Terpstra test.

Table 2 presented the demographic and tongue features of the study participants. The prevalence of comorbidities increased significantly with worsening renal, whereas 27.0% of the CKD stage 3 group and 31.1% of the CKD stage 4–5 group were comorbidities with diabetes (*p* < 0.001). Besides, 19.1% of the CKD stage 3 group and 17.5% of the CKD stage 4–5 group were companied with hypertension (*p* < 0.001). Participants with wet saliva were significantly more likely to exhibit decreased eGFR, with an increasing trend observed as renal function decreased (*p* < 0.001). The color and thickness of tongue fur showed significant correlations with CKD severity. Yellow fur and thick fur were more prevalent in participants with lower eGFR (*p* < 0.001). Pale tongue color was associated with poorer renal function, being more common in CKD stage 3 and stage 4–5 groups (*p* < 0.001).

**Table 2:** Demographic and tongue diagnosis features of study participants with statistical comparisons across CKD stages.

		**eGFR** ≧**60 (*****n*** = **318)**		**30** ≦**eGFR**<**60 (*****n*** = **345)**		**eGFR**<**30 (*****n*** = **103)**		***P*-value**
		***N***	**%**	***N***	**%**	***N***	**%**	
Gender	Female	168	52.8	187	54.2	55	53.4	0.826
	Male	150	47.2	158	45.8	48	46.6	
Diabetes mellitus	No	318	100.0	252	73.0	71	68.9	< 0.001
	Yes	0	0.0	93	27.0	32	31.1	
Hypertension	No	318	100.0	279	80.9	85	82.5	< 0.001
	Yes	0	0.0	66	19.1	18	17.5	
Saliva	Normal	230	72.3	192	55.7	54	52.4	< 0.001
	Wet	88	27.7	153	44.3	49	47.6	
Fur color	White	239	77.9	115	35.0	36	36.7	< 0.001
	Yellow	68	22.1	214	65.0	62	63.3	
Tongue color	Pale	44	13.8	128	37.1	44	42.7	< 0.001
	Pink	186	58.5	170	49.3	47	45.6	
	Red	66	20.8	14	4.1	5	4.9	
	Bluish	22	6.9	33	9.6	7	6.8	
Tongue shape	Small	176	55.3	192	55.7	46	44.7	0.456
	Median	49	15.4	71	20.6	24	23.3	
	Enlarged	93	29.2	82	23.8	33	32.0	
Fur thickness	Thin	214	67.3	157	45.5	46	44.7	< 0.001
	Thick	99	31.1	180	52.2	53	51.5	
Little fur	No	247	78.9	292	86.6	93	93.9	< 0.001
	Yes	66	21.1	45	13.4	6	6.1	

*P*-value by chi-square test for trend.

Table 3 shows the results of the ordinal logistic regression analysis, which was conducted to identify factors associated with the stage of CKD. Each additional red dot on the tongue was associated with a decrease in the severity of CKD, with an ordinal odds ratio (OR) of 0.972 (95% CI: 0.965–0.979, *p* < 0.001), while each additional tooth mark was associated with a reduction in the severity of CKD, with an OR of 0.938 (95% CI: 0.884–0.995, *p* = 0.032). The presence of ecchymoses on the tongue was positively associated with the stage of CKD, with an OR of 1.031 (95% CI: 1.007–1.056, *p* = 0.012). Participants with wet saliva had a significantly higher likelihood of severe CKD, with an OR of 2.536 (95% CI: 1.805–3.564, *p* < 0.001). Yellow tongue fur was strongly associated with increased stage of CKD, with an OR of 3.195 (95% CI: 2.281–4.477, *p* < 0.001). Pale tongue color was associated with increased severity of CKD, with an OR of 2.107 (95% CI: 1.445–3.072, *p* < 0.001). Bluish tongue color was also associated with increased severity, with an OR of 2.743 (95% CI: 1.516–4.962, *p* = 0.001). Red tongue color was not significantly associated with CKD severity (*p* = 0.542).

**Table 3:** Ordinal logistic regression analysis of tongue diagnosis features and clinical factors associated with eGFR severity.

**Variables**		**Estimate**	**SE**	**Ordinal odds ratio**	**95% CI**	***P*-value**
Red dots		−0.029	0.004	0.972	0.965–0.979	< 0.001
Ecchymoses		0.031	0.012	1.031	1.007–1.056	0.012
Teeth marks		−0.064	0.030	0.938	0.884–0.995	0.032
Saliva	Wet	0.931	0.174	2.536	1.805–3.564	< 0.001
	Normal	0.000		1.000		
Fur color	Yellow	1.162	0.172	3.195	2.281–4.477	< 0.001
	White	0.000		1.000		
Tongue color	Pale	0.745	0.192	2.107	1.445–3.072	< 0.001
	Red	−0.259	0.425	0.772	0.336–1.775	0.542
	Bluish	1.009	0.302	2.743	1.516–4.962	0.001
	Pink	0.000		1.000		
Diabetes mellitus	Yes	1.135	0.207	3.110	2.074–4.665	< 0.001
	No	0.000		1.000		
Hypertension	Yes	0.748	0.234	2.113	1.336–3.341	0.001
	No	0.000		1.000		

The thresholds for CKD severity were defined as follows.

**Stage 0**: eGFR ≥ 60 ml/min/1.732 (control group, no CKD).

**Stage 1**: 30 ≤ eGFR < 60 ml/min/1.732 (CKD Stage 3).

**Stage 2**: eGFR < 30 ml/min/1.732 (CKD Stage 4–5, without dialysis).

## 4 Discussion

In this study, we employed an ATDS, which provides an objective and standardized method to capture and analyze tongue features. This minimizes the subjectivity and variability found in traditional tongue diagnosis. Previous research on tongue diagnosis in TCM has laid a solid foundation by exploring associations between tongue features and various diseases, including metabolic syndrome, type 2 diabetes, ischemic stroke, and breast cancer. For example, Chung et al. (2023) identified several tongue characteristics, including fur thickness, color, ecchymosis, teeth marks, and red dots, as potential markers of renal function in CKD patients (Chung et al., 2023). However, the study was limited by a small sample size. In this case-control study, we utilized data from a previous study to identify 340 healthy participants and enrolled 481 patients with chronic kidney disease (CKD) who underwent TCM tongue diagnosis. The use of ATDS minimized the subjective assessments typically made by practitioners, thereby reducing inconsistencies in diagnosis.

Statistical analyses revealed that specific tongue features were significantly associated with renal function severity. Hemoglobin, ecchymoses, teeth marks, and red dots were inversely related to renal function, decreasing as renal function worsened. Conversely, thick tongue fur and wet saliva increased with worsening renal function. The ordinal logistic regression analysis identifies several significant predictors of CKD severity as measured by eGFR. Tongue features such as red dots, ecchymoses, teeth marks, fur color, and tongue color are significantly associated with eGFR severity, demonstrating the diagnostic potential of these non-invasive markers. Additionally, clinical conditions such as diabetes mellitus and hypertension are confirmed as significant predictors of severe CKD. These findings suggest that the ATDS can provide valuable, objective data for the early detection and monitoring of CKD, aligning TCM practices with modern diagnostic methodologies.

According to the TCM theory, tongue diagnosis involves diagnosing a disease by observing changes in the tongue body and tongue fur of a patient. In clinical practice, tongue diagnosis is a highly valuable method for determining the strength of vital force (i.e., *qi*), the depth of the disease location, the nature of pathogens, and the changes in symptoms. In TCM, a pale tongue is equivalent to anemia in Western medicine. Anemia occurs in 34–94.3% CKD patients, mainly due to decreased renal erythropoietin, iron, folic acid, or vitamin B12 deficiency, poor erythrocyte survival, and blood loss during dialysis (Tajbakhsh et al., 2013; Tajalli et al., 2021; Gluba-Brzozka et al., 2020). Wet saliva in TCM corresponds to abnormal water metabolism in the body in Western medicine. Wet saliva also indicates spleen deficiency syndrome in TCM, which hinders thorough blood filtration and transpiration of excess water, causing edema in patients with kidney disease (Wu et al., 2020). According to TCM theory, the spleen plays an important role in various physiological functions, including regulating the transport and metabolism of water and nutrients in the body (Wu, 1998). When the spleen is incapable of transporting body fluids, it leads to an accumulation of moisture within the body, a condition referred to as water dampness, which signifies spleen-deficiency syndrome in TCM (Yang and Jia, 2013).

When the tongue appears to be yellow and heavily coated, this indicates damp-heat syndrome in TCM, which is caused by a combination of dampness and heat (Yuan et al., 2021). According to TCM theory, excess saliva is caused by spleen-deficiency syndrome; yellow thick tongue coating is often related to damp-heat syndrome (Zhou et al., 2022; Kang et al., 2022). Yellow fur color usually results from poor oral hygiene as food and bacteria can collect on the filiform papillae on the surface. In TCM theory, a yellow tongue fur indicates an interior heat pattern. In CKD patients, yellow fur may correspond to the accumulation of uremic toxins and metabolic waste products that the kidneys are unable to filter out, such as BUN and creatinine. Several studies have discussed the relationship between CKD and tongue characteristics such as tongue-coating thickness, tongue-coating microbiota, and metabolic markers (Guo et al., 2022; Chung et al., 2023).

TCM classifies these vascular complications as blood stasis. Tongue diagnosis plays a key role in TCM, where ecchymosis and engorged sublingual collateral vessels are manifestations of blood stasis. Numerous diseases can cause blood stasis, including cardiovascular disease, cerebral vascular accidents, and CKD (Park et al., 2015; Kouri and Rheault, 2021). Patients with CKD are at high risk of developing ESRD, cardiovascular disease (associated with arterial hypertension), and cardiovascular mortality (Sun et al., 2014).

One study reported that patients with CKD commonly exhibited features such as blood deficiency and stasis with qi deficiency (Wang et al., 2022). Our study indicated that patients with the later stages of CKD had more yellow thick fur compared to the control group. This suggested that damp-heat syndrome may also be a clinical characteristic of CKD in the context of TCM. In modern medicine, renal failure results from kidney injury, leading to reduced renal function, which causes electrolyte dysregulation and the accumulation of fluids, body waste, and toxins. Severe kidney dysfunction can lead to uremic syndrome (Makris and Spanou, 2016). According to TCM theory, uremic syndrome is related to asthenia and excess superficiality. Excess syndrome is characterized by ecchymoses, red dots, and thick white or yellow fur, while deficiency syndrome is characterized by teeth marks, pale tongue, and excess saliva. In general, TCM generally believes that spleen-kidney deficiency is an internal condition where blood deficiency, blood stasis, dampness, and heat are interconnected with the patient's viscera. Although no significant differences in teeth marks were observed, there was a trends toward disease progression, and teeth marks are often seen in dialysis patients (Rashpa et al., 2018).

This study has certain limitations. First, this was a cross-sectional study. The cross-sectional nature of this study captures data at a single point in time, which limits the ability to establish causality between tongue features and the progression of CKD. Secondly, underlying conditions or medication use might influence tongue features independently of CKD. Although we adjusted for key comorbidities like diabetes and hypertension in our statistical models, we recognize that other factors, including medication use, might introduce bias. Third, this study excluded patients undergoing dialysis, who often present with distinct tongue features due to their advanced disease state and treatment effects. The longitudinal study designs and more diverse and broader patient population would provide a more robust understanding of how tongue features change over time and in relation to CKD progression. Fourth, we did not analyze sublingual collateral vessels, which might offer additional insights into vascular complications related to CKD. Future studies should consider including these features for a more thorough assessment.

## 5 Conclusion

In conclusion, tongue features such as paleness, wet saliva, yellow fur, and ecchymosis were prevalent in CKD patients and may serve as early clinical indicators of the CKD. This study demonstrates that TCM tongue diagnosis, facilitated by ATDS, is a valuable, non-invasive method for identifying CKD and distinguishing its stages.

## Data Availability

The data supporting the findings of this study are available from the corresponding author on request.
